# Single Calcite Particle Dissolution Kinetics: Revealing the Influence of Mass Transport

**DOI:** 10.1021/acsmeasuresciau.2c00025

**Published:** 2022-07-12

**Authors:** Xinmeng Fan, Christopher Batchelor-McAuley, Minjun Yang, Richard G. Compton

**Affiliations:** † Physical and Theoretical Chemistry Laboratory, Department of Chemistry, University of Oxford, South Parks Road, Oxford OX1 3QZ, Great Britain

**Keywords:** Calcite dissolution kinetics, Optical Microscopy, Diffusion limited flux, Mass transport control, Carbonate Speciation, Interfacial kinetics

## Abstract

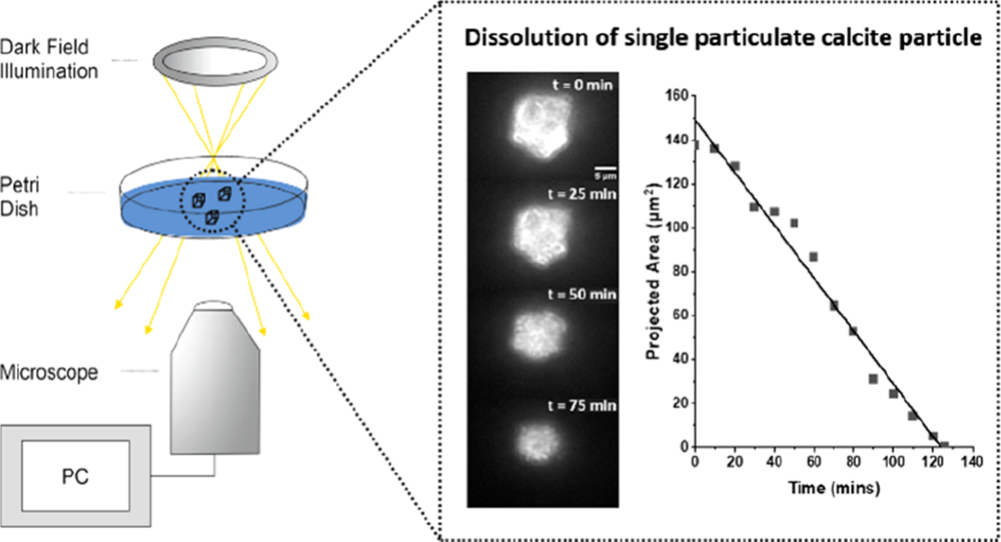

Calcite dissolution kinetics at the single particle scale are determined. It is demonstrated that at high undersaturation and in the absence of inhibitors the particulate mineral dissolution rate is controlled by a saturated calcite surface in local equilibrium with dissolved Ca^2+^ and CO_3_^2–^ coupled with rate determining diffusive transport of the ions away from the surface. Previous work is revisited and inconsistencies arising from the assumption of a surface-controlled reaction are highlighted. The data have implications for ocean modeling of climate change.

## Introduction

In the ocean, there are essentially two principal pH buffering systems. In the solution phase the carbonic acid system dominates the control of the water pH, such that limited additions of an acid or base lead to changes in the homogeneous inorganic carbon speciation.^1^ Further, the water is also in contact with solid carbonate minerals where dissolution of the mineral acts as a heterogeneous buffering mechanism.^2^ The buffering capacity of the heterogeneous system is significantly larger than the homogeneous process.^3^ Ultimately, the fate of anthropogenically formed CO_2_ is the reaction of the gas with calcium carbonate to form solution phase bicarbonate in the oceans.^4^ Although the calcite dissolution reaction is both well studied^5^ and of global significance, there is a lack of hydrodynamically well-defined and reproducible ways to study the dissolution of micrometer sized particles. Generally, to study the dissolution of small calcite particles, the release of material from a suspension is monitored as a function of time.^6^ The problem here, as highlighted in the 1980s by Sjoberg and Rickard,^7−9^ is that the hydrodynamic conditions near the surface of the particle are not well-defined such that these experiments can only be used to yield qualitative insight into the interfacial dissolution rates and these results are not easily applied to understanding the process under different hydrodynamic conditions.^7^ Fundamentally, calcite dissolution corresponds to the formation of calcium and carbonate ions as expressed below:^1^
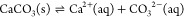
1Calcite is only sparingly soluble with a solubility product of 3.3 × 10^–9^ M^2^ (25 °C),^10^ but a major complicating factor here is that carbonate is a base (p*K*_a_ 10.3); hence, under aqueous conditions, the solubility of the solid varies with the acid/base properties of the solution.

At near neutral pH (>6), with low carbon dioxide concentrations and in the absence of calcium and carbonate ions in the solution phase (high undersaturation), it is generally accepted^5^ that the calcite dissolution rate is ∼10^–6^ mol m^–2^ s^–1^ at 25 °C. This dissolution reaction under such conditions is often taken as one of the fundamental pathways for calcite dissolution, and a rate of 1.19 × 10^–6^ mol m^–2^ s^–1^ has been compiled for use in the modeling of natural water systems by the U.S. Geological Survey.^11^ This value originates from work by Plummer, Wigley, and Parkhurst who, in 1978, studied the dissolution rate of suspensions of calcite particles in the range of 300–600 μm and at 25 °C.^6^ Further, using similar experimental techniques and employing a suspension of particles, this kinetic measurement has, over the course of half a century, been ‘validated’ and corroborated by a number of different researchers and groups, as evidenced by the none exhaustive list of literature examples provided in Table 1.

**Table 1:** Literature Examples of Calcite Dissolution Rate at pH > 6 and High Undersaturation^a^

particle size/μm	rate/mol m^–2^ s^–1^	ref
300–600	1.19 × 10^–6^	(6)
90–125	6.91 × 10^–7^	(12)
300–400	6.5 × 10^–7^	(13)
600	1.82 ± 0.2 × 10^–6^	(14)
70–100	3.8 × 10^–6^	(15)
1–10	2.8 × 10^–6^	(16)
single macroscopic crystal (∼cm)	2.34 × 10^–6^	(17)

a Note the sizes do not necessarily directly reflect the particle size in the solution phase where agglomeration can be a significant factor.

For any interfacial reaction, either the surface reaction rate can be controlled by the rate of reaction at the surface itself or it can be controlled by the mass transport of material to/from the interface. The data as reported in Table 1 relates to a surface reaction flux. Importantly, the fact that the rates presented in Table 1 are all comparable does not provide good evidence that these measured rates reflect the true interfacial reaction rate. If we assume an equilibrium concentration of calcium ions of 0.1 mM, a diffusion coefficient of 1 × 10^–9^ m^2^ s^–1^, and a diffusion layer thickness of ∼100 μm, then the expected mass transport limited rate is ∼1 × 10^–6^ mol m^–2^ s^–1^. The expected mass transport limited rate for this reaction is of the same order of magnitude as those reported in Table 1 for the calcite surface reaction rates! Furthermore, Sjoberg and Rickard demonstrated in the 1980s that for a *macroscopic single crystal* the interfacial reaction rate is in reality significantly higher, where for Iceland Spar^9^ they reported a rate of 1.41 × 10^–5^ mol m^–2^ s^–1^ and for Carrara marble^8,9^ an even higher rate of 4 × 10^–5^ mol m^–2^ s^–1^ was determined. Away from such single crystal experiments, the physical difficulty in understanding the mass transport regime for particle suspensions arises from both the uncertainty in the contribution of convection to the transport of material to/from the mineral surface of larger particles and the potential for particles to agglomerate and/or sediment depending on the used conditions. Particle agglomeration/aggregation in the solution phase leads to the effective particle size being significantly larger, which in turn decreases the mass transport rate to/from the mineral interface.

The dissolution of particulate calcite, as opposed to macroscopic slabs of material is of global signficance. This wider relevance stems from the fact that, in the oceanic environment, of the order of 10^15^ grams of calcite is biogenically precipitated at the surface of the world’s oceans per annum.^18^ This calcite is predominantly formed by calcifying single cellular plants such as microscopic coccolithophores which encrust themselves in protective calcite plates. This biogenic calcite is present as micrometer-sized particles as it traverses down the water column. Of this material formed at the ocean’s surface layer, only about 30% will be deposited as sediments and the remaining particulate material dissolves as it sinks to the depths of the ocean.^19^ Understanding these carbon fluxes and the chemistry that underpins them is imperative for gaining insight into how the oceanic environment will respond to increasing levels of atmospheric CO_2_. The literature reported dissolution kinetics for crystalline particulate calcite materials (see Table 1) are in stark contrast with recent work by Hassenkam et al., who used individual particles connected to an atomic force microscopy (AFM) tip to monitor calcite dissolution.^20^ Under deionized water conditions, they report dissolution rates approximately an order of magnitude larger (∼1 × 10^–5^ mol m^2^ s^–1^). Note in this literature work, by reporting the rate in units of amount per area per time, the authors are again implicitly assuming that the rate is controlled by the interfacial reaction kinetics. This paper therefore poses the questions: in the case of small particulate calcite material, as is of direct relevance in the environment, how fast are the interfacial dissolution kinetics, can they be measured under a well-defined hydrodynamic regime, and what controls the dissolution rate? The answers are of profound importance to ocean modeling as well as of fundamental significance.

In this work, we present a simple optical microscopy experiment that enables the dissolution of individual micrometer sized calcite particles to be monitored under, as will be demonstrated, a well-defined mass transport regime. A further major advantage of studying this reaction at the single particle scale is that the mass transport to and from the micrometer scale particle is very rapid, potentially enabling the resolution of fast interfacial reaction kinetics. Further, studying the reaction at the single particle scale partially reflects the situation in the oceans where microscopic particles are dissolved as they descend through the water column. Notably the attained single particle mass transport rates are comparable to those produced using a macroscopic disc rotating at 10,000 rpm. Using these high mass transport rates, for the first time, we prove that under near neutral conditions and at high undersaturation the dissolution of small calcite particles is, for the used material, controlled not by the interfacial reaction rate but by the mass transport flux of products away from the interface.

## Experimental Section

### Materials

The deionized water used was MilliQ ultrapure water with a resistivity of 18.2 MΩ cm at 25 °C. NaHCO_3_ and Na_2_CO_3_ were bought from Acros Organics, and CaCl_2_ was purchased from Aldrich. The pH of the solution was adjusted with HCl (Fisher chemical) and NaOH (Honeywell).

The calcite particles used in the experiments were synthesized via precipitation from a mixture of supersaturated calcium chloride and disodium carbonate solutions. Large quantities of calcium chloride and disodium carbonate salt were separately added to two vials containing 10 mL of deionized water until saturation was reached. The two saturated solutions were then mixed, and a precipitate of the calcium carbonate formed. The solution containing the precipitate was left for 3 days to ensure the material was calcite and then diluted with saturated CaCO_3_ solution to reduce the number density of the calcite particles. The saturated CaCO_3_ solution contained 1 mM CaCl_2_ and 10 mM NaHCO_3_ and was adjusted to pH 8 with HCl and NaOH. Then 10 μL of the original precipitate solution was diluted with 2 mL of the saturated CaCO_3_ solution. For the experiments in this work, 10 μL of the diluted solution was added to 20 mL of deionized water.

### Optical Microscopy Measurements

A 20× objective (Olympus UPLXAPO 20x, Olympus Corporation, Tokyo Japan) was used for the optical measurements. The dark-field illumination was applied via a LED Illuminator (Aura Pro Phase Contrast Illuminator, Cairn Research, Kent U.K.), and FLIR Blackfly S camera (BFS-U3-88S6M-C, Teledyne FLIR LLC, U.S.) acquired the images every 60.0 s. The images provided were 8 bit and gray with an exposure time of 1 ms.

The images were analyzed by using ImageJ freeware (Fiji). The projected area of each calcite particle in pixels is determined by thresholding the edge manually with the Freehand Selection Tool and measuring the selected area in pixels. The actual projected area is the number of pixels in the 2D image multiplied by the pixel resolution (0.155 × 0.155 μm^2^ pixel^–1^). The side length of the calcite cube is calculated by the square root of projected area.

### Scanning Electron Microscopy

A Sigma 300 FEG-SEM from Zeiss was used to obtain the scanning electron microscopy (SEM) images with an 8.0 kV accelerating voltage.

### X-ray Powder Diffraction (XRD)

XRD diffractograms were collected on a Bruker D8 Advance diffractometer with a LynxEye detector and Cu Kα1 radiation (λ = 1.5406 Å), operating at 40 kV and 25 mA (step size at 0.019°, time per step at 0.10 s, total number of steps at 4368), unless specified. Samples in powder form were pressed onto a glass preparative slide which was then attached to a sample holder. All measurements were scanned at 2θ of 5–90°.

## Results and Discussion

**Figure 1:**
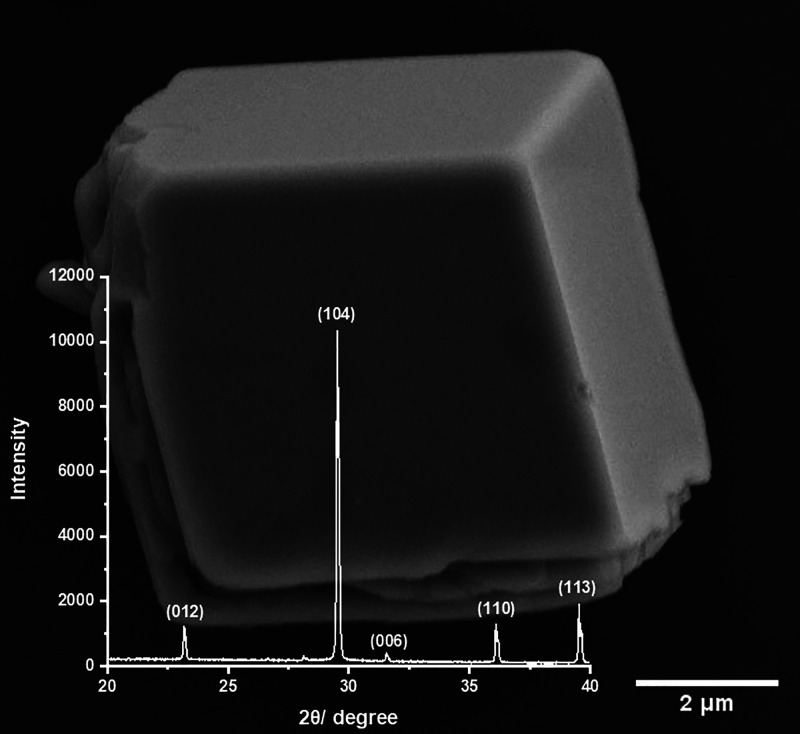
Characterization of the CaCO_3_ precipitate. The gray cube is the SEM image of an exemplar cuboid CaCO_3_ precipitate. The white spectrum is the XRD pattern showing the specific reflection for calcite (hR10, *R*3̅*c* (167)) with *d*-spacings/Å: 3.83, 3.02, 2.83, 2.48, 2.28; corresponding to *hkl*: 012, 104, 006, 110, 113, respectively, which is identical to the spectrum reported by Rodriguez-Blanco et al.^21^ for calcite.

In this work, the particles of calcite used were synthesized directly from the precipitation of a supersaturated solution made from the mixing of calcium chloride and disodium carbonate solutions; full information is provided in the Experimental Section. An example SEM image of a representative calcite particle is depicted in Figure 1, showing the rhombohedral shape. Also overlaid on Figure 1 is the X-ray powder diffraction pattern of the material confirming the material to be pure calcite. From this precipitation method, the particles were found to have side lengths in the range of 4–16 μm. It has previously been reported that this precipitation method initially leads to the formation of amorphous calcium carbonate which over the course of hours is converted into calcite;^21^ hence, after precipitation of the calcium carbonate, the material was left for 3 days prior to experimentation to ensure the material was calcite. In this work, we opted to not use a commercial calcite sample as SEM images revealed the material to be composed of aggregates of smaller crystallites sintered together (see Supporting Information (SI) section 1).

**Figure 2:**
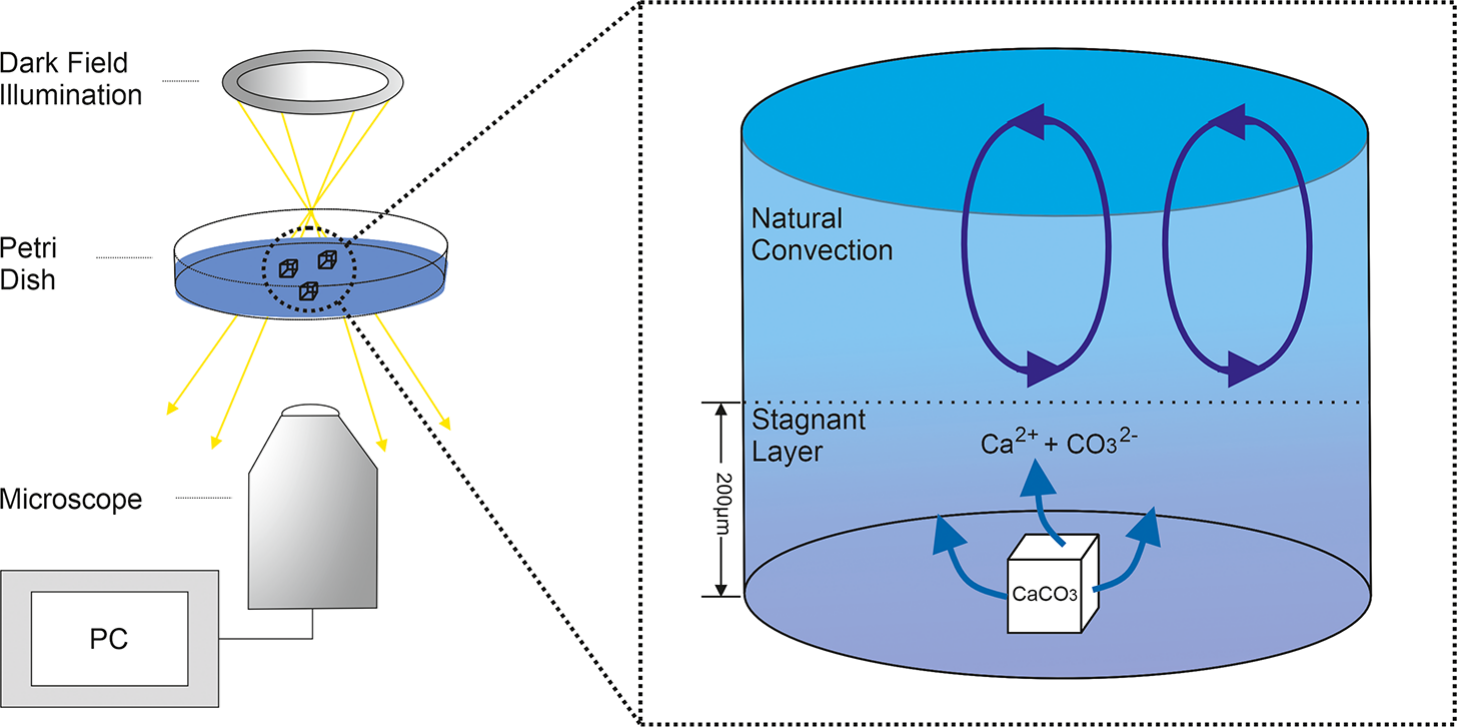
Schematic of the dark-field microscopy experiment setup.

An inverted microscope was used to visualize the calcite particles. The calcite particles were placed in a Petri dish such that they were submerged and had ∼10.0 mm of aqueous solution above them. This thick layer of solution was used to ensure that bulk ion concentrations were not significantly altered by the dissolution of the calcite. Figure 2 provides a schematic diagram of the instrumentation used where a ring of LEDs illuminates the calcite sample at an oblique angle to provide a dark-field setup. The particles are situated on the lower glass surface and imaged from below using a 20× objective (Olympus). A time lapse video of the particles dissolving is subsequently recorded allowing the dissolution process to be monitored and, as will be demonstrated, accurately quantified.

An important point about this experimental setup is that although in the bulk solution there will be significant convective motion of the solution, near the glass surface this movement is heavily damped. As recognized by Nernst,^22^ the damping of the convective flow near a solid surface means that in the vicinity of the glass substrate of the Petri- dish the mass transport will be essentially a diffusion only process.^23^ The extent of this stagnant “diffusion-only” layer adjacent to a large planar surface has been previously experimentally investigated^24^ and shown to extend some 200–250 μm away from the planar surface.^25,26^ This damping of the bulk convection has some important and useful implications for the present work. First, a stagnant layer of solution will exist in the vicinity of the glass surface. Second, as long as the calcite particles are sufficiently small, the mass transport can, with high accuracy, be considered a diffusion only process due to the shielding from the bulk convective motion afforded by the glass surface. The question of, how large a particle can be before convective motion to the structure needs to be considered, is important. However, on the basis of work in the literature, it is concluded to be reasonable that if the particles of study are less than 40 μm^27^ in diameter then the flux to the mineral interface will be well described by using a diffusion only model. In this work, the largest particle we consider has a lateral dimension of 15.6 μm. This experimental reduction of the system to being a diffusion only process such that the geometry of the system is that of “a particle on a plate” is important. The lower complexity of the problem enables quantitative assessment of the mass transport regime to and from the particle surface.

**Figure 3:**
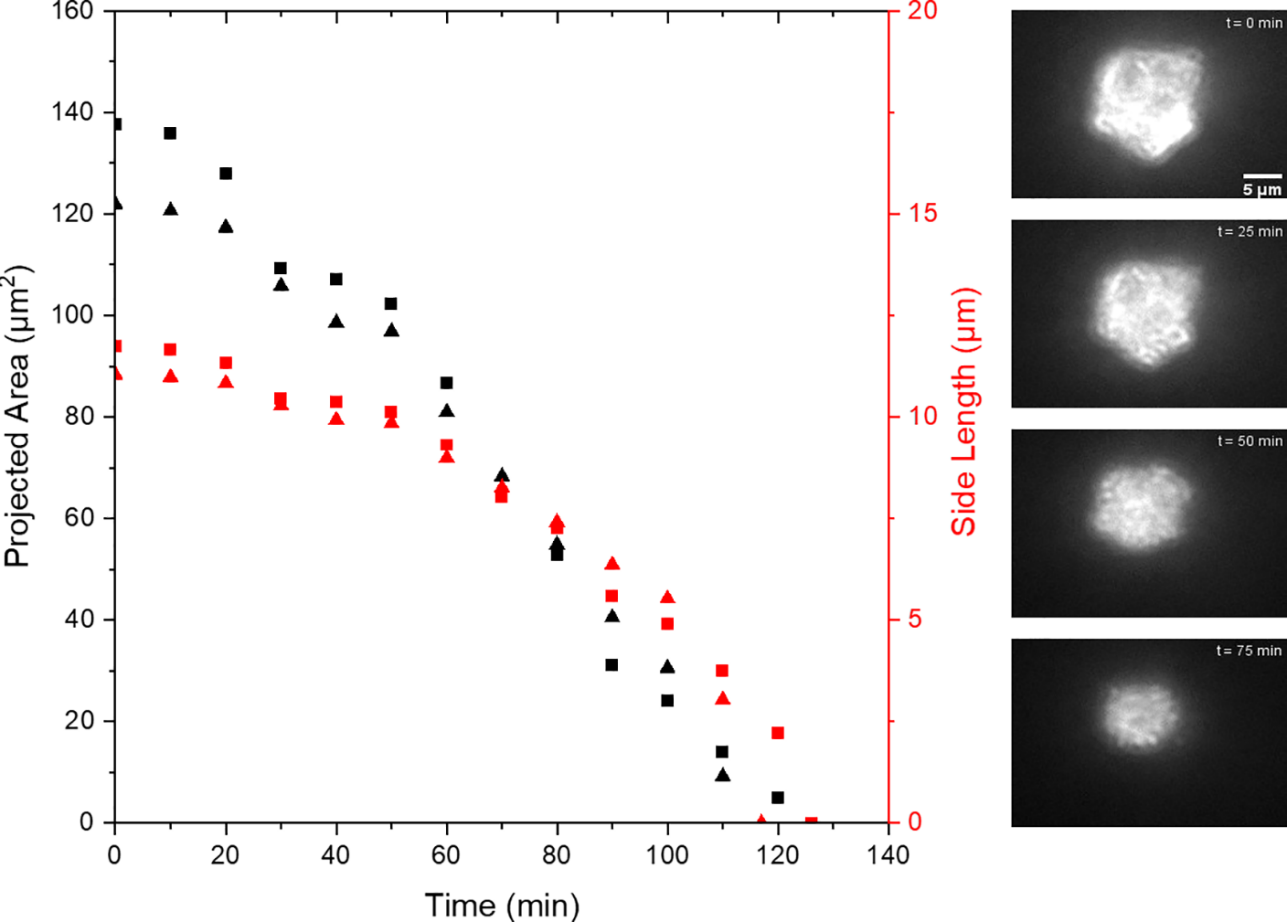
Measured projected area of the calcite precipitate in DI water as a function of time. The right-hand side shows the set of optical images of a 137.7 μm^2^ particle changing with time. On the left-hand side, it is the plot of area and the square root of the area (side length of an equivalent cube) changing with time. Black represents area, and red represents side length. The square markers represent a particle with an initial area of 137.7 μm^2^ which dissolved in 126 min. The triangle markers represent a different particle which had an initial area of 122.0 μm^2^ and dissolved in 117 min.

A 10.0 μL sample of calcite particles was injected into a 20.0 mL deionized water sample contained in a Petri dish. The calcite sample contained approximately 1.1 × 10^–7^ moles of calcium carbonate, and hence, even after dissolution, the bulk composition of the water sample was not significantly altered (<1% of the equilibrium ion concentration). Figure 3 presents a dark-field microscope image of a single particle as it dissolves over the course of 2 h. A microscope image of the particles was recorded every 60.0 s. From this series of images, the projected area of the particle was measured during the course of the dissolution process. In the following, as shown in Figure 1, the particles are initially approximately rhombohedral in structure; however, as seen in Figure 3, the geometry of the particles does change to some extent over the course of the dissolution process. Nevertheless, for simplicity, we assume that the particles are at all stages of dissolution quasi-cuboidal. Hence, Figure 3 presents the projected areas of two different particles in a deionized water sample. Also plotted is the effective side length of the particle (the square-root of the projected area) where we have assumed the particle to be cuboidal in structure.

In the case that the dissolution reaction is controlled by the rate of the interfacial reaction, the variation of the particle side length (*L*/m) as a function of time (*t*/s) is expected to be
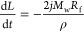
2where *j* is the flux density (mol m^–2^ s^–1^), *M*_w_ is the calcite molecular weight (100.1 g mol^–1^), ρ is the density of calcite (2.71 × 10^6^ g m^–3^),^28^ and *R*_f_ is the roughness factor, a dimensionless value that is the ratio of the particle’s actual surface area relative to its geometric surface area. The factor of 2 reflects the fact that the particle is being dissolved from both sides of the cube. Consequently, if the flux density is a constant, as would be expected for a surface limited reaction, and the surface roughness does not change significantly, then d*L*/d*t* is expected to be a constant.

Figure 3 demonstrates that the experimentally measured particle side lengths clearly do not vary linearly as a function of time and the rate of dissolution (d*L*/d*t*) increases as the particle decreases in size. SI section 2 provides the linear best fit of this data showing that the linear fit to this side length data has an average R^2^ value of 0.89.

In contrast to the above situation, if the dissolution reaction is controlled by the transport of material to or from the particle then the expected flux (*j*/mol m^–2^ s^–1^) can be approximately described by the following:^29^
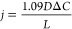
3where *D* is the species diffusion coefficient (m^2^ s^–1^), Δ*C* is the difference in the equilibrium concentration as compared to the bulk (Δ*C*/mol m^–3^ = *C*_eq_ – *C*_bulk_), and *L* is the side length of the cube (m). This expression (eq 3) comes from the previously reported total flux to a cubic particle on a surface, as has been determined numerically, divided by the total active surface area of the particle 5*L*^2^. Note this lower surface area (as compared to 6*L*^2^) reflects the fact that one face of the cube is not diffusionally accessible due to it being blocked by the supporting substrate. In eq 3 we are making the approximation that the diffusional flux density is uniform across the particle surface. Note in the SI section 3 we also analyze the particles on the assumption that they are quasi-spherical, this different geometric assumption only changes the expected flux by less than 1%. From substitution of eq 3 into eq 2, the change in the particle side length as a function of time, for a diffusion limited reaction, is expected to be
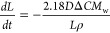
4Note for a diffusion limited reaction we do not need to consider the particle roughness, *R*_f_, as the mass transport rate is proportional not to the particles surface area but to the geometric size of the object. Integration of expression (eq 4) allows the variation in the particle projected area as a function of time to be analytically expressed, giving:
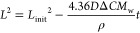
5where *L*_init_ is the initial side length of the particle. Importantly, *L*^2^ is equal to the 2D projected area of the particle as measured by the microscope. Herein we refer to this measured 2D area as the projected area of the particle. Consequently, on the basis of eq 5, it can be seen that, for a *diffusion limited* dissolution of the calcite particle, the particle’s projected area (as for example measured by an optical microscope) is expected to decrease linearly as a function of time. Figure 3 plots the projected areas for two calcite particles in deionized water, as measured optically, as a function of time and demonstrates that the projected area of the particle decreases essentially linearly with time (see SI section 2 for the linear best fit of this data). For these two particles that have lateral dimensions of approximately 10 μm in this deionized water sample, the complete dissolution of the material takes approximately 2 h. Hence, given the linearity of the dissolution rate, we can express the dissolution rate (*k*) in units of area per time. From the measurement of three particles, the average dissolution rate of this calcite material in deionized water is found to be 1.7 ± 0.1 × 10^–14^ m^2^ s^–1^. The above provides some evidence, on the basis of the variation of the particle size as a function of time during the dissolution reaction, for the dissolution process being diffusion limited. It is however plausible that the data presented in Figure 3, showing the nonlinearity of the plot of d*L*/d*t*, could be due to some other factor, for instance, a change in mechanism as the dissolution process proceeds. However, as will be explored below the rates predicted through eq 5 are, within error, those recorded experimentally, providing strong evidence for and corroborating the conclusion that the calcite dissolution reaction for this calcite particles is at the mass transport limit.

Having experimentally determined the dissolution rate of the calcite particles in deionized water (1.7 ± 0.1 × 10^–14^ m^2^ s^–1^), on the basis of eq 5, what is the theoretically expected dissolution rate based on a diffusion limited model? To apply eq 5, the equilibrium calcite dissolution concentration and hence Δ*C* needs to be assessed.

To calculate the equilibrium concentrations of the solution phase species, the following six equations need to be solved:
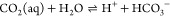
6
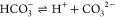
7
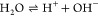
8
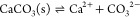
9
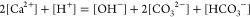
10
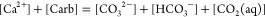
11where [Carb] is the total inorganic carbon in the system due to equilibration of the water with the atmosphere. Eqations 6–9 are the solution phase equilibria for the carbonate/calcite system, and the associated thermodynamic constants have been reported to a high degree of accuracy across a range of temperatures and salinities^10^ (for further information see SI section 4). Beyond these four equilibria, two additional auxiliary equations are required; eq 10 is an expression of electroneutrality, and eq 11 is a conservation of mass expression that reflects the stoichiometry of the calcite solid. SI section 5 outlines how this system of equations can be solved iteratively. Under DI water conditions, where the water initially contains 13.1 μM of inorganic carbon ([Carb]) from the atmosphere and is at a pH of 5.7 (see the detailed calculation in SI section 6), the calcite solubility is 0.114 and 0.123 mM at 18 and 25 °C, respectively. In the present work, where the calcite particles are dissolved in a Petri dish, the temperature of the solution is not perfectly controlled. However, from measurements taken during the course of the experiments, it was found that the solution temperature was in all cases in the range of 18–25 °C. Note this ambient temperature range is significantly larger than the temperature changes expected to occur due to the dissolution reaction. From the enthalpy of solution and the heat capacity of water, the temperature at the interface of the calcite particle is estimated to be altered by less than 1 K. Given that the temperature affects the calcite solubility and the associated diffusion coefficients, this uncertainty in the temperature represents a significant uncertainty in the analysis. At room temperature, a decrease of 7 °C raises the calcite solubility product by ∼9%. Note the increase in the solubility of the calcite as a function of temperature (0.114 to 0.123 mM) is driven by the decrease in the associated p*K*_a_’s. Furthermore, the associated diffusion coefficients will drop by around 20%. Going forward, all of the theoretical results will be given as a range representing the values expected for these two temperatures.

Using the above solubility calculations, we can use eq 5 to provide a direct estimate of the diffusion only mass transport limited dissolution rate. Here we assume that the diffusion coefficient is equal to the geometric mean of the calcium and carbonate diffusion coefficients (7.25 × 10^–10^ and 8.70 × 10^–10^ m^2^ s^–1^ at 18 and 25 °C). Importantly, this simple model contains no adjustable parameters. Assuming the veracity of the input parameters, the accuracy of the model only depends on the approximations made in its derivation. Under DI water conditions, the theoretically expected diffusion limited dissolution of a cuboidal particle on a flat surface is in the range of 1.33–1.72 × 10^–14^ m^2^ s^–1^, which compares with the experimentally measured value of 1.7 ± 0.1 x10^–14^ m^2^ s^–1^. We conclude that under these conditions the dissolution rate is fully consistent with the reaction being at the diffusion limit. Assuming that the reaction is fully controlled by the rate of mass transport of material away from the mineral interface, a lower bound on the true heterogeneous rate constant can be estimated. If we take the lower temperature range values for both *D* and Δ*C* and if we assume that the reaction remains under diffusion control until at least a projected area of 50 μm^2^, as evidenced by Figure 3, then using eq 3, a lower limit for the heterogeneous dissolution rate can be set at >1 × 10^–5^ mol m^–2^ s^–1^. This number, although significantly larger than those generally previously reported in the literature (ca. 1 × 10^–6^ mol m^–2^ s^–1^), should be compared to the previously measured rates of 1.41 × 10^–5^ and 4 × 10^–5^ mol m^–2^ s^–1^ for Iceland Spar^9^ and Carrara Marble,^8,9^ respectively. It does not seem unreasonable for the small (micrometer sized) crystallites, as used in this work, to potentially exhibit higher dissolution rates than these two macroscopic crystal surfaces given the likely higher surface coverage of defects. Further, on this basis, we conclude that the data presented in Table 1 likely predominantly reflect the prevailing mass transport conditions as opposed to giving an accurate measurement of the interfacial reaction kinetics. For larger particles or when using macroscopic crystal surfaces, the diffusion limited flux is significantly lower than that which occurs at the single particle scale, hence leading to the lower measured dissolution rates. From a geological perspective, the dissolution of calcite under higher ionic strength conditions is also of relevance; SI section 7 presents data for the dissolution rate up to an ionic strength of 1 M NaCl.

To the best of the authors’ knowledge, the only previous article in which the calcite dissolution from *individual* calcite particles has been studied is in the work of Hassenkam et al.^20^ who investigated the dissolution of both inorganic and biogenic calcite under deionized water conditions. This was achieved by attaching the calcite particles to an AFM cantilever tip and monitoring the change in particle mass as a function of time. In this work, they do not relate their measurements to those previously made in the literature; moreover, they analyze their data on the basis of the reaction being controlled by the interfacial kinetics of the dissolution reaction. Furthermore, as noted by the authors themselves the measurement procedure is “...extremely time consuming and very tricky”, thus limiting the general applicability of their approach. However, they report dissolution rates in deionized water that are of the order of 1 × 10^–5^ mol m^–2^ s^–1^, and further, the measured dissolution rate seems to vary as a function of the particle size. If this data presented by Hassenkam et al. is reanalyzed (see SI section 9 for details) assuming the calcite material dissolves at a mass transport dissolution rate, then this literature data is consistent with a measured dissolution rate of 1.35–1.55 × 10^–14^ m^2^ s^–1^. Note the work by Hassenkam et al. was performed at 23 °C using a calcite particle attached to an AFM cantilever tip. Notably the mass transport regime to such a tip supported particle is not well-defined. Consequently, there is ∼30% uncertainty in the expected diffusional mass transport limited dissolution rate (this is further discussed in SI section 9). However, on the basis of an analogous calculation to that reported above, we predict that in this literature work the expected dissolution rate is in the range of 1.3–2.1 x10^–14^ m^2^ s^–1^. This is in excellent agreement with the dissolution rate inferred from their reported data (1.35–1.55 × 10^–14^ m^2^ s^–1^). Consequently, in both experiments reported in this work and that reported in the literature by Hassenkam et al., under deionized water conditions, the calcite dissolution rate is consistent, within the accuracy of the presented analysis, with it being at the mass transport limit. As a further point of interest, the reported dissolution rates for both a cultured coccolith and a fossilized lith as reported in this literature work are also consistent with being at the mass transport limit when the dissolution was studied in deionized water.

In summary in the absence of inhibitors and under deionized water conditions, the interfacial kinetics of the calcite dissolution rate of small mineral particulates are not measurable and the reaction is controlled by the mass transport of ions in the solution phase. It is inferred that the work in the literature that has reported far lower dissolution rates for calcite particles and powder samples has, in some cases, been performed under hydrodynamic conditions where the dissolution flux is controlled by mass transport away from a surface locally in equilibrium with CaC*O*_3_(*s)*. In this case, the reaction is predominantly described by the following:
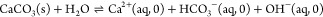
12
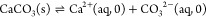
13where 0 indicates that the species is formed at the mineral surface. In the present case, these surface species are in equilibrium with the solid carbonate material. The rate-determining step is then the flux of these ions into the bulk solution. In principle, if the interfacial kinetics of the calcite dissolution process are to be resolved, then the reaction likely needs to be studied with smaller particles, for example, submicro- or even nanoparticles, and hence under higher mass transport conditions. Experimentally studying the dissolution of such smaller particles may necessitate the use of more advanced optical techniques, for example, iSCAT,^30^ to ensure that the dissolution of the smaller particles to be resolved.

## Conclusions

In deionized water, the dissolution of small calcite particles is shown to be at the mass transport limit for the flux of the solution phase ions away from the mineral interface. For the material used in this work, the heterogeneous dissolution rate is >1 × 10^–5^ mol m^–2^ s^–1^ in deionized water. This measurement has been enabled by using a bespoke optical system that allows the dissolution of individual and isolated micrometer sized particles to be monitored and for the reaction to be studied under diffusion only conditions. As the particle dissolves the projected area of the particle decreases at an experimentally determined rate of 1.7 ± 0.1 x10^–14^ m^2^ s^–1^. The experimental use of a diffusion only system allows the mass transport limited flux to be analytically predicted using a model that contains no unknowns or fitting parameters. From the calculation of the calcite equilibrium speciation, the diffusional mass transport limited dissolution is found to be 1.33–1.72 × 10^–14^ m^2^ s^–1^ (18–25 °C). The close agreement of the theoretically predicted and experimentally measured dissolution rates indicates that the process is, within the accuracy of this analysis, at the mass transport limit. More generally, the importance of mass transport in controlling the calcite dissolution rate has, under some conditions, not been fully recognized in the literature. Given the global relevance of this heterogeneous process, these results may have significant implications from both a fundamental and applied perspective.

## Supporting Information Available

SEM image of commercial calcite; linear best fit of dissolution data in DI water; theoretically predicted rate based on spherical geometry; equilibria constants as a function of temperature and salinity; concentration of carbonate species from the atmosphere in deionized water; chemical equilibria solved in log-space; calcite dissolution as a function of ionic strength; reanalysis of the literature data (PDF)
