# Supplementary material for: Single Calcite Particle Dissolution Kinetics: Revealing the Influence of Mass Transport
Source: ACS Meas Sci Au. 2022 Jul 12;2(5):422–9. doi: 10.1021/acsmeasuresciau.2c00025 (PMC9885995; doi:10.1021/acsmeasuresciau.2c00025)
Supplement: Supplementary file 1 — tg2c00025_si_001.pdf [file tg2c00025_si_001.pdf]

Supporting Information: Single calcite particle dissolution kinetics: revealing the influence of mass-transport

Xinmeng Fan[1], Christopher Batchelor-McAuley[1], Minjun Yang[1], Richard G. Compton[1*]

[1] Physical and Theoretical Chemistry Laboratory, Department of Chemistry, University of Oxford, South Parks Road, Oxford OX1 3QZ, Great Britain.

* corresponding author email: Richard.Compton@chem.ox.ac.uk

Section 1: SEM image of an exemplar commercial calcite aggregate

Commercial calcite powders tend to be comprised of sintered aggregates of calcite crystallites. Figure S1 presents a representative SEM image of one such aggregate particle. In the present work knowledge of the mass-transport regime is imperative to evidence that the reaction is under diffusive control. Consequently, this precludes the use of such irregularly shaped material in the present work.

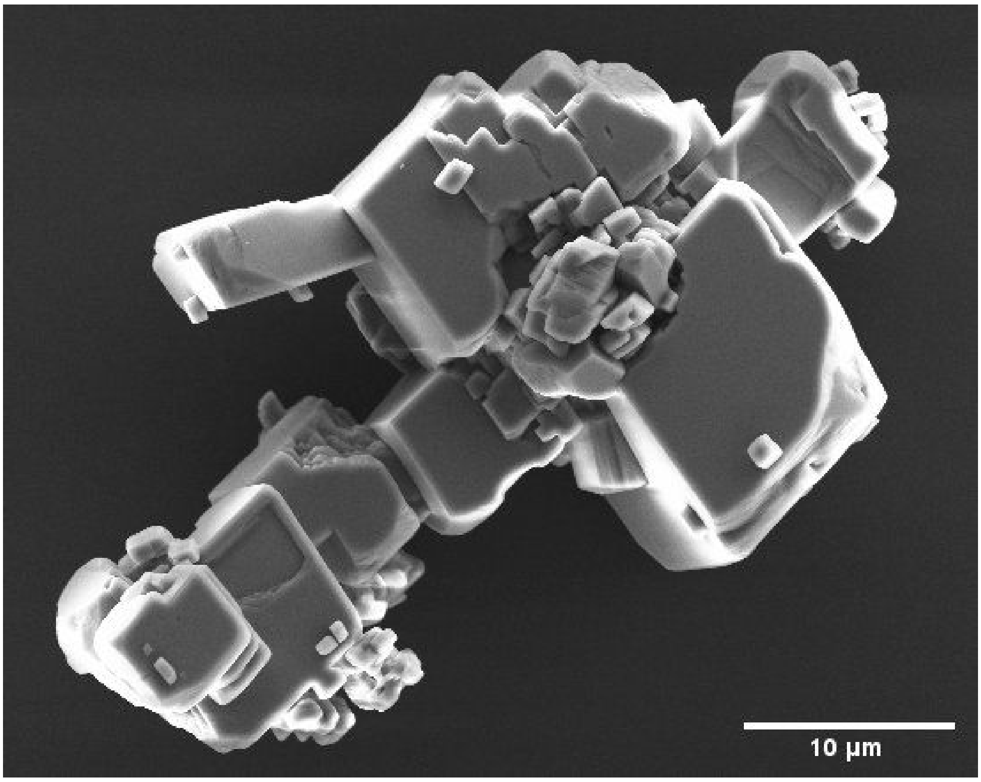

Figure S1: SEM image of an exemplar commercial calcite aggregate

Section 2: Linear best fit of calcite precipitate dissolved in DI water

As shown in Figure S2, the linear best fits for the projected areas have an R^2 of 0.98 and 0.96 for the two particles presented. Whereas, the linear best fit to the side length is lower with an R^2 0.93 and 0.84.

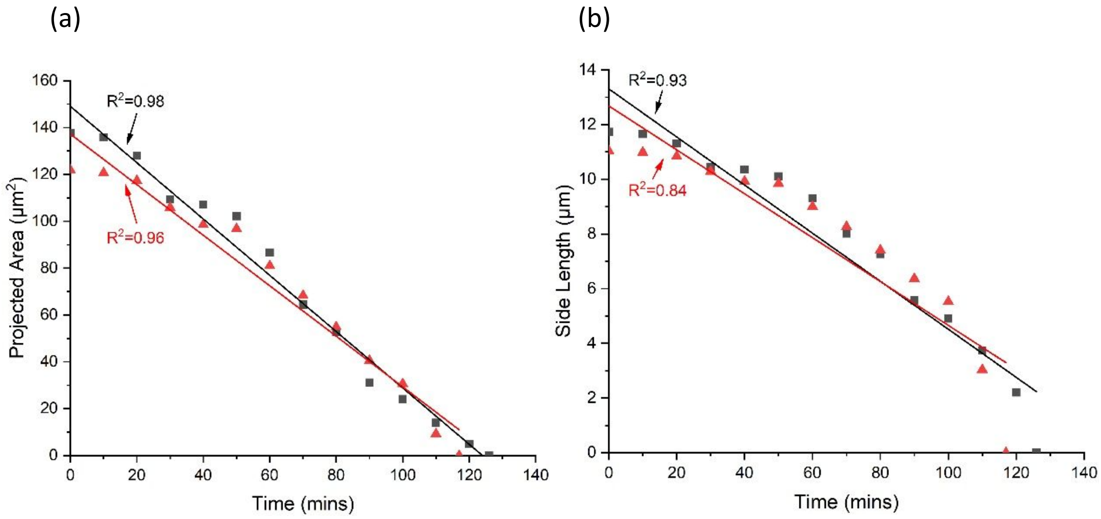

Figure S2: Linear best fit of (a) projected area and (b) side length as a function of time. Black cube and red triangle represent two different calcite precipitates.

Section 3: Analysis based on quasi-spherical shape

In the main body of the text we analyse the dissolution rate on the assumption that the particle can be described as being quasi-cuboidal in structure. As an alternative approximation we also derive the expected dissolution flux for a spherical particle on a surface. As will be demonstrated the change in the assumption regarding the geometry of the particle does not significantly alter the quantitative results.

Derivation of the dissolution rate (k) in units of area per time

For a sphere on a surface, the expected diffusional flux (j /mol m^{-2} s^{-1}) is [1]:

$$j = \frac{4\pi D\Delta C r \ln(2)}{4\pi r^2} = \frac{D\Delta C \ln(2)}{r} \qquad (1)$$

D is the species diffusion coefficient (m^2 s^{-1}), ΔC is the difference in the equilibrium concentration as compared to the bulk ($\Delta C/\mathrm{mol\,m}^{-3} = C_{eq} - C_{bulk}$) and r is the effective radius of the sphere (m). Based on equation (1), the change in effective radius can be presented as:

$$\frac{dr}{dt} = -\frac{D\Delta C Mw * \ln(2)}{r\rho} \qquad (2)$$

where M_w is the molecular weight (100.1 g mol^{-1}) and ρ is the density of the material (2.71x10^6 g m^{-3}). Integration of equation (2) gives an expression for change in the projected area as a function of time:

$$4\pi r^2 = 4\pi r_{init}^2 - \frac{2\ln(2)\pi D\Delta C M_w}{\rho} t \qquad (3)$$

This expression is very similar to that presented in equation (3) of the main text, where the predicted rates differ by less than 1% of each other.

Section 4: Equilibrium constant and variation with ionic strength and temperature

There are 4 equilibrium constants to be considered:

1. Solubility product of calcite K_{sp}: $CaCO_3(s) \rightleftharpoons Ca^{2+} + {CO_3}^{2-}$
2. Effective first acid dissociation K_{a1}: $CO_2(aq) + H_2O \rightleftharpoons H^+ + {HCO_3}^-$
3. Second acid dissociation K_{a2}: ${HCO_3}^- \rightleftharpoons H^+ + {CO_3}^{2-}$
4. Water self-ionisation K_w: $H_2O \rightleftharpoons H^+ + OH^-$

These constants vary with both the ionic strength and temperature. Here we report the values reported in the literature that are used to quantify these thermodynamic paramters. First, ionic strength (I) is converted to salinity (S) via the equation[2] below:

$$S = \frac{1000I}{19.920 - 1.0049I}$$

For K_{sp}, the equations of Mucci[3] are used:

$$\log K_{sp}^{*} = \log K_{sp}^{0} + AS^{0.5} + BS + CS^{1.5}$$

$$A = -0.77712 + 0.0028426T + \frac{178.34}{T}$$

$$B = -0.07711, C = 0.0041249$$

$$\log K_{sp}^{0} = -171.9065 - 0.077993T + \frac{2839.319}{T} + 71.595 \log T$$

For K_{a1} and K_{a2}, the equations from Millero *et al*[4] are used:

$$pK_{a_i}^{*} - pK_{a_i}^{0} = A_i + \frac{B_i}{T} + C_i \, lnT$$

$$pK_{a_1}^{0} = -126.34048 + \frac{6320.813}{T} + 19.568224 \, lnT$$

$$pK_{a_2}^{0} = -90.18333 + \frac{5143.692}{T} + 14.613358 \, lnT$$

$$A_1 = 13.4191S^{0.5} + 0.0331S - (5.33 \times 10^{-5})S^2$$

$$B_1 = -530.123S^{0.5} - 6.103S$$

$$C_1 = -2.06950S^{0.5}$$

$$A_2 = 21.0894S^{0.5} + 0.1248S - (3.687 \times 10^{-4})S^2$$

$$B_2 = -772.483S^{0.5} - 20.051S$$

$$C_1 = -3.3336S^{0.5}$$

For K_w, the equation from Dickson and Riley[5] is used:

$$pK_w = \frac{3441.0}{T} + 2.256 - 0.709\, I^{0.5}$$

Section 5: Chemical equilibria solved in log-space

In the main body of the text equations 4-9 are a set of 6 equations that need to be solved for simultaneously to find the equilibrium concentrations of the aqueous species. Where the solution phase concentrations of Ca^{2+}, CO_3^{2-}, HCO_3^-, CO_2, H^+ and OH^- are unknowns to be found.

As noted in the text the thermodynamic solution phase equilibria (equations 4-7) have been reported to a high degree of accuracy as a function of temperature and salinity.[2](See Section 4) The relevant constants are defined as follows:

$$K_{a1} = \frac{[\mathrm{H}^+][\mathrm{HCO_3}^-]}{[\mathrm{CO_2}]} \quad (4)$$

$$K_{a2} = \frac{[\mathrm{H}^+][\mathrm{CO_3}^{2-}]}{[\mathrm{HCO_3^-}]} \quad (5)$$

$$K_w = [\mathrm{H}^+][\mathrm{OH}^-] \quad (6)$$

$$K_{sp} = [\mathrm{Ca}^{2+}][\mathrm{CO_3}^{2-}] \quad (7)$$

here we use the convention of using stoichiometric constants.[2] By using concentrations as opposed to activities the thermodynamic constants vary as a function of the composition of the solution. Further we have taken the activity of both calcite and water to be unity such that they do not appear in the above thermodynamic expressions. Using the above 4 equations in combination with the two auxiliary equations presented in the main text we then have six unknowns and six simultaneous equations. Hence the only challenge is finding a solution for this non-linear set. Generally this would be approached using the Newton-Raphson multi-dimensional root finding method.[6] In the present case, the Newton-Raphson method was found to be very sensitive to the initial guess and did not always converge to a result. However, the use of a Newton-Raphson method in log space is for more efficient for this type of problem.[7] Taking the log of thermodynamic expressions 4-7 converts them from being multiplicative to a simple addition. The problem is how to deal with the auxiliary equations (8 and 9 in the main text); logging a sum does not yield a simple expression. One way forward is to re-express the auxiliary equations as a geometric mean using the knowledge that the arithmetic mean is always greater than or equal to the geometric mean. Making this approximation allows the equation set to be expressed as a set of approximately correct linear equations which are to be solved.[7] Although this log space method involves more algebraic manipulation the principal advantage is the relative insensitivity of the result to the initial guess.

Section 6: Concentration of inorganic carbon ([Carb]) from the atmosphere in deionised water

At 18°C and in the absence of calcite, the equations need to be solved for are:

1. $CO_2(aq) + H_2O \rightleftharpoons H^+ + HCO_3^-$
   Effective First Acid Dissociation $Ka_1 = 4.02 \times 10^{-7}$ M

2. $HCO_3^- \rightleftharpoons H^+ + CO_3^{2-}$
   Second Acid Dissociation $Ka_2 = 4.01 \times 10^{-11}$ M

3. $H_2O \rightleftharpoons H^+ + OH^-$
   Water Self-ionisation $Kw = 8.42 \times 10^{-15}$ M^2

4. $[H^+] = [HCO_3^-] + 2[CO_3^{2-}] + [OH^-]$
   Bulk electroneutrality

where the equilibrium constant is calculated based on the equations in SI section 4. There are 2 knowns and 4 unknowns for this system. The 2 knows are [CO_2 (g)] and [CO_2 (aq)], which are 1.41×10^{-5} M and 1.09×10^{-5} M respectively. 4 unknowns are [HCO_3^-], [CO_3^{2-}], [H^+] and [OH^-]. The detailed calculation for the 2 unknows is shown below:

$$[CO2(g)] = \frac{1}{22.4} \times 10^{-3.5} \times 1 \times 10^3 = 1.41 \times 10^{-5}\, M$$

$$\frac{[CO_2(aq)]}{[CO_2(g)]} = 0.77, so [CO_2(aq)] = 1.09 \times 10^{-5}\, M$$

Based on the method presented in the SI section 5, we can solve this set of chemical equilibria and find the 4 unknowns yielding:

$$[HCO_3^-] = 2.09 \times 10^{-6}\, M, [CO_3^{2-}] = 4.00 \times 10^{-11}\, M$$

$$[H^+] = 2.09 \times 10^{-6}\, M, [OH^-] = 4.03 \times 10^{-9}\, M$$

Hence,

$$[Carb] = [CO_2(aq)] + [HCO_3^-] + [CO_3^{2-}] = 13.1\, \mu M$$

$$pH = -\log_{10}[H^+] = 5.7$$

Where the pH here has been defined on a concentration basis, given the low ionic strength of the solution the proton activity coefficient will be essentially unity and hence the actual pH will also have a value close to 5.7.

Section 7: Calcite dissolution as a function of ionic strength

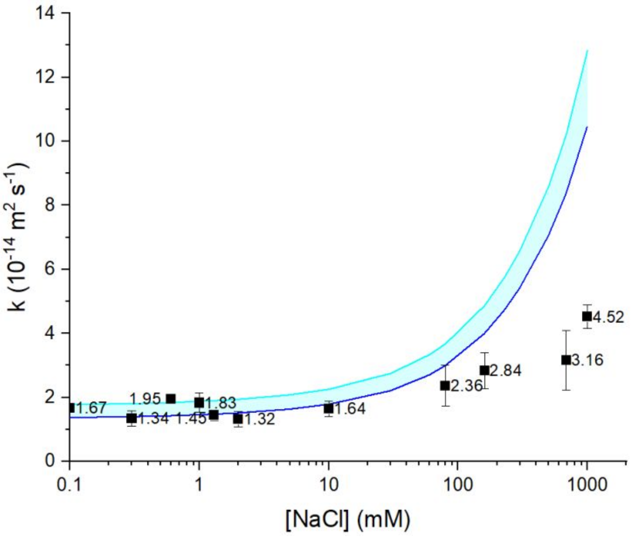

Figure S3: Experimental and expected dissolution rate of $CaCO_3$ in DI water as a function of [NaCl]. Scattered points (black) are the experimental dissolution rate, which is projected area divided by dissolution time. The band overlaid is the expected rate based on a diffusion limited model for a cuboidal shape ($= \frac{4.36 D \Delta C M_w}{\rho}$) – dark blue line represents 18°C and pale blue line represents 25°C.

From a geological perspective the dissolution of calcite is important in both fresh and seawater conditions. Potable water generally has an ionic strength of 10 mM whereas the ionic strength of seawater is far higher at ~0.7M. The solution phase ionic strength has an appreciable effect on the solution phase thermodynamics of the calcite system. Most notably at near seawater ionic strength conditions the concentration product (the stoichiometric calcite solubility product) increases by almost two orders of magnitude in the high salt content. Here we consider how the dissolution rate varies as a function of the ionic strength. Using the same experimental setup as outlined in the main text the dissolution of individual calcite particles were investigated in aqueous NaCl solutions up to an ionic strength of 1M. Figure S3 plots the measured calcite dissolution rate as a function of the ionic strength of the solution. As can be seen an increase in the ionic strength leads to essentially no change in the dissolution rate up to approximately 10 mM NaCl. However, at higher concentrations the experimental dissolution rate increases by approximately a factor of two. Where at 1 M NaCl the dissolution rate is measured to be 4.5×10^{-14} m^2 s^{-1}. Also overlaid on Figure S3 is the theoretically expected dissolution rate as a function of ionic strength. The diffusion only predicted rates and the experimentally measured values are in good agreement at low ionic strength, but at concentrations above 10 mM there is a divergence. Where at the highest ionic strength of 1 M the predicted rate based on a model of diffusion control is approximately three times greater than measured

experimentally. Conventionally this lower measured dissolution rate might be interpreted as evidencing the dissolution rate to be under a mixed kinetic regime and partially under control of the heterogeneous rate constant. However, challengingly and as shown in the SI section 8 (see below) there is little evidence in the plot of the particle's projected area as a function of time to suggest that the dA/dt is significantly deviating away from linearity. This implies that the reaction may still be under mass-transport control even at higher ionic strengths.

What may be the cause of this rate of the interfacial reaction as a function of the ionic strength? The thermodynamic expressions (equations 8-11) used to calculate the equilibrium concentrations of the solution phase species can be usefully rearranged to give the following thermodynamically equivalent reaction scheme:

$$\mathrm{CaCO_3(s)} \rightleftharpoons \mathrm{Ca^{2+}} + \mathrm{CO_3}^{2-} \quad (8)$$

$$\mathrm{CaCO_3(s)} + \mathrm{H^+} \rightleftharpoons \mathrm{Ca^{2+}} + \mathrm{HCO_3}^{-} \quad (9)$$

$$\mathrm{CaCO_3(s)} + \mathrm{H_2O} \rightleftharpoons \mathrm{Ca^{2+}} + \mathrm{HCO_3}^{-} + \mathrm{OH^-} \quad (10)$$

$$\mathrm{CaCO_3(s)} + \mathrm{CO_2(aq)} + \mathrm{H_2O} \rightleftharpoons \mathrm{Ca^{2+}} + 2\mathrm{HCO_3}^{-} \quad (11)$$

These four reactions do not represent elementary reaction processes but reflect the overall thermodynamic reactions involved in the dissolution process. Depending on the experimental conditions, notably in the present case the changing ionic strength of the solution, the relative contribution and weighting of each of these reactions will change. By considering the change in equilibrium calcite speciation it is possible to directly assess the contribution of each reaction process to the total dissolution rate.

As the ionic strength of the aqueous solution is increased the calcite solubility increases by a factor of ~4. What we wish to evidence though is that although the solubility is increasing the relative contributions of reaction 8-9 also change as a function of ionic strength. The amount of calcite that can be dissolved in the system is calculated by solving equations 4-9 of the main text. By studying the resulting expected speciation, it is possible to determine to what extent equations 8-9 contribute to the overall dissolution of the calcite material. For instance, under deionised water conditions at 25°C, the equilibrium speciation is:

$[Ca^{2+}]$ = 0.114 mM

$[CO_2(aq)]$ = 28.8 nM

$[HCO_3^-]$ = 95.4 µM

$[CO_3^{2-}]$ = 31.6 µM

$[H^+]$ = 0.12 nM

$[OH^-]$ = 69.6 µM

From these concentrations it is possible to determine the relative weightings of equation 8-11; however, in the case where dissolved inorganic carbon is already present in the solution then the changes in concentration need to be considers. Due to the stoichiometry of the calcite dissolution reaction, $[Ca^{2+}]$ equals the total amount of calcite that can be dissolved. Hence the relative contributions of each pathway are given by:

Equation (8):

$\Delta[CO_3^{2-}] / \Delta[Ca^{2+}]$ = 27.6%

Equation (9):

$-\Delta[H^+] / \Delta[Ca^{2+}]$ = 1.8%

Equation (10):

$\Delta[OH^-] / \Delta[Ca^{2+}]$ = 61%

Equation (11):

$-\Delta[CO_2] / \Delta[Ca^{2+}]$ = 9.5%

It is possible to track the contribution of these different reaction pathways as a function of ionic strength as shown in Figure S4. Figure S4 plots the relative contributions of these 4 thermodynamic processes to the calcite dissolution as a function of the system's ionic strength. First, under deionised water conditions then equations (9) and (11) only contribute minimally to the overall dissolution process (<10%). Conversely, reactions (8) and (10) are thermodynamically far more important and for each carbonate ion dissolved approximately two-thirds will be protonated resulting in the associated formation of a hydroxide ion whereas the remaining third will be present as carbonate. The increasing ionic strength alters this speciation pattern most notably the increase in the calcite solubility product means that a higher proportion of the material results in the formation of carbonate as opposed to bicarbonate ions.

As can be seen in Figure S3 this switch in the reaction scheme occurs at the 10 mM ionic strength level. This change in the reaction pathway may tentatively give some indication as to the physical reasons

why the calcite dissolution reaction rate deviates away from the simple mass-transport limit at higher ionic strengths.

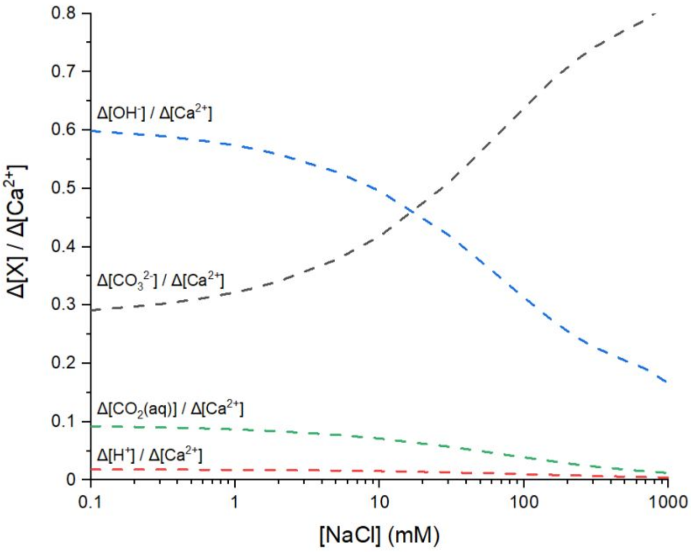

Figure S4: Change in theoretically predicted calcite dissolution pathway as a function of the ionic strength of the aqueous solution. Each curve represented the relative contribution of the different thermodynamic pathways, red; proton dissolution (reaction 9), green; carbon dioxide dissolution (reaction 11), black; direct dissociation (reaction 8) and blue; dissolution via reaction with water (reaction 10).

Section 8: Measured projected area of calcite in 1M NaCl

As discussed in detail in the SI section 7, the dissolution of the calcite particles was also studied at higher ionic strength. Here we present representative data for the data measured at an ionic strength of 1 M NaCl. As with the data in the main body of the text the experiment is to optically monitor the dissolution of a calcite particle, Figure S5 shows three representative images of a particle over the course of 20 minutes as it dissolved. Further Figure S5 also plots the measured projected area and equivalent particle side length (square root of the projected area). As with the data measured at a low ionic strength dA/dt seems to be linear and dL/dt is not a constant. Consequently, we conclude that at high ionic strength, the dissolution rate does not appear to be under a mixed kinetics regime.

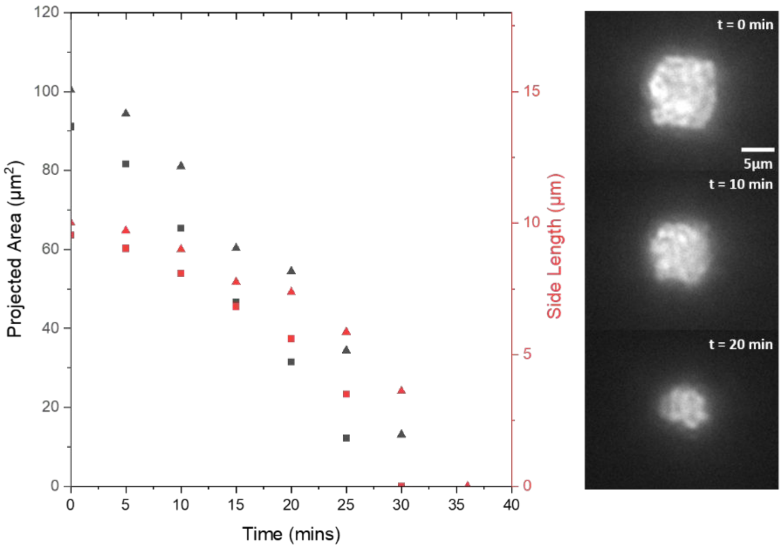

Figure S5: Measured projected area of calcite precipitate dissolved in 1M NaCl. On the right-hand side, it is the set of optical images of a 91.1 μm² particle changing with time. On the left-hand side, it is the plot of area and side length changing with time. Black represents area and red represents side length. Square is a particle with an initial area of 91.1 μm² and dissolved in 30 mins. Triangle is another particle with an initial area of 100.4 μm² and dissolved in 36 mins.

Section 9: Reanalysis of the literature data

The literature data presented by Hassenkam *et al.*[8] reports the mass of a calcite particle as a function of dissolution time. This measurement was made by attaching a calcite particle to an AFM cantilever tip and determining the weight of the particle as a function of time as it dissolves. They studied the dissolution rates in both deionised water and calcium free artificial seawater. Herein we will only focus on the analysis of their deionised water data; the presence of magnesium ions in the artificial seawater will have an influence on the dissolution rate.[9]

Due to the geometry of the used system (particle on a tip) in this literature work the mass-transport regime is not as well defined as the system reported in this present article. Specifically, the calcite particle is away from a surface and the question arises as to what extent the mass-transport to/from the mineral interface is blocked by the cantilever tip. Either the particle can be viewed as isolated in solution or on a surface. For a cubic particle in solution the mass-transport limited flux[10] (J / mol s^{-1}) is:

$$J = \frac{16.7 D\Delta C L}{2} \qquad (8)$$

whereas for a cube on a surface the flux[10] is:

$$J = \frac{10.9 D\Delta C L}{2} \qquad (9)$$

The lower rate relates to the blocking effect of the surface, for comparison in the case of a sphere the presence of the surface decreases the flux by a factor of ln(2).

For a cuboidal particle the mass of the particle is equal to:

$$Mass = \rho L_{final}^{3} \qquad (10)$$

Hence,

$$\frac{dM}{dL} = 3L^2\rho \qquad (11)$$

Consequently, for a particle isolated in solution we have the expression:

$$\frac{dL}{dt} = -\frac{16.7 D\Delta C Mw}{6L\rho} \qquad (12)$$

Consequently,

$$Mass = \rho L^3 = \rho\left(L_{initial}^{2} - \frac{5.57 D\Delta C Mw}{\rho} t\right)^{3/2} \qquad (13)$$

Similarly, for a cube on a surface then:

$$Mass = \rho L^3 = \rho({L_{initial}}^2 - \frac{3.63D\Delta CMw}{\rho}t)^{3/2} \quad (14)$$

For the AFM experiment, the temperature is fixed at 23 °C. Assuming D varies linearly between 18-25 °C, then the geometric average D of Ca^{2+} and $CO3^{2-}$ is 8.28×10^{-10} m^2s^{-1}, and the difference in the equilibrium dissolved inorganic carbon concentration is ΔC of 0.121 mM. Hence, for a calcite cube in DI water, the expected dissolution rate is in the range of 1.34-2.06 $\times 10^{-14}$ m^2s^{-1}.

Synthetic calcite crystals in DI water

Using the expression

$$Mass = \rho {L_{final}}^3 = \rho({L_{initial}}^2 - kt)^{3/2} \quad (15)$$

the data presented by Hassenkam *et al.* can be graphically re-analysed to find the dissolution rate for the calcite material presented in this literature work. For a quasi-cuboidal calcite crystal, 4.11ng mass is equivalent to 11.5μm side length. 4.11ng is calculated from initial mass 4.44ng subtracting the non-calcite mass 0.33ng. Using Eq (15) with the converted initial side length, mass versus time is plotted with different k values. As shown in Figure S6, for their experiment, the best fit value of k is in the range of 1.35-1.55$\times 10^{-14}$ m^2 s^{-1} (blue line), this is in excellent agreement with the theoretical value calculated in this work (1.34-2.06 $\times 10^{-14}$ m^2 s^{-1} green dashed lines).

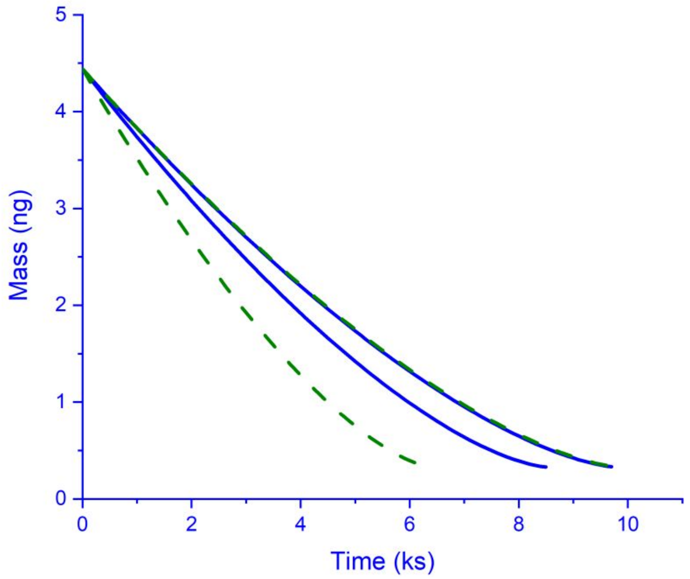

Figure S6: Fitting of the data presented by Hassenkam et al. solid blue line represents the best fit of equation 15 to the literature data, where the data is consistent with a rate in the range of 1.35-1.55x10^{-14} m^2 s^{-1}. Dashed green line represents the range of rates predicted by equations 13 and 14. Here ks is kiloseconds (10^3 s). This figure should be directly compared to Figure 2A in Hassenkam, T., et al. "Tracking single coccolith dissolution with picogram resolution and implications for CO2 sequestration and ocean acidification." Proceedings of the National Academy of Sciences 108.21 (2011): 8571-8576.

Cultured and fossilised coccolith in DI water

In the work of Hassenkam *et al.* they also present the measured dissolution rates for both cultured and fossilised liths. Here we analyse these biogenic calcite particles assuming that the mass-transport is that of a cube (as above) and show that again for this biogenic calcite the dissolution rate is consistent with being at the mass-transport limited rate.

Using the same calculation as used for the synthetic calcite, 358.06pg (= 458.06 – 100pg) is converted to 5.1µm side length for cultured coccolith and 681.25pg (=850 – 168.75pg) is converted to 6.3µm side length for fossilised coccolith. The result is shown in Figure S7. The best fit k is in a range of 1.00-1.80x10^{-14} m^2 s^{-1} (blue lines), which is comparable to the synthetic calcite rate reported above.

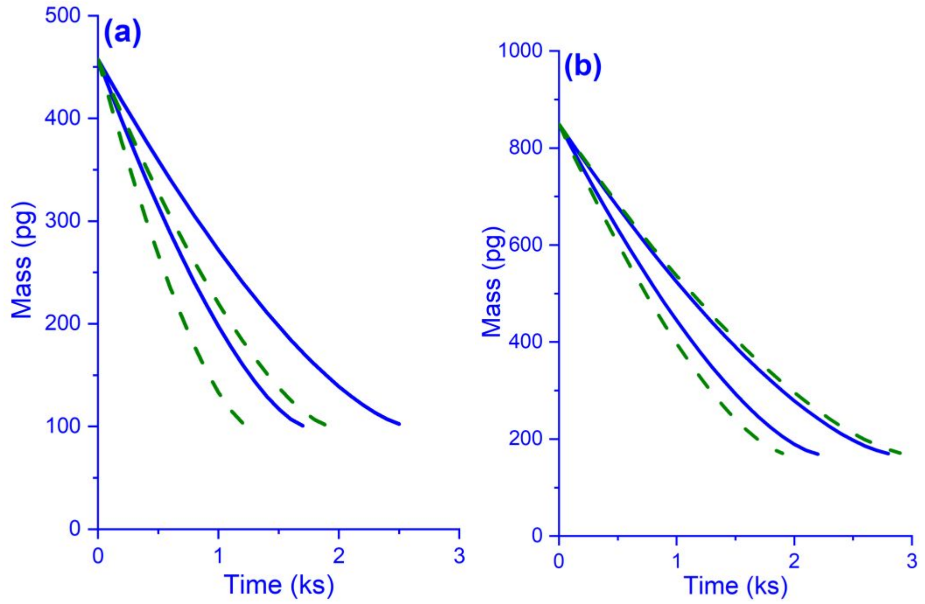

Figure S7: Mass loss of (a) cultured coccolith and (b) fossilised coccolith in DI water using equation 15 to fit the data provided in Figures 3A and 3D in Hassenkam, T., et al. "Tracking single coccolith dissolution with picogram resolution and implications for CO2 sequestration and ocean acidification." *Fitted k (solid blue line) and theoretical k (dash green line). The best fit k is in the range 1.00-1.50x10^{-14} m^2 s^{-1} for (a) cultured coccolith and 1.40-1.80x10^{-14} m^2 s^{-1} for (b) fossilised coccolith. Here ks is kiloseconds (10^3 s).*

References

(1) Bobbert, P. A.; Wind, M. M.; Vlieger, J. Diffusion to a slowly growing truncated sphere on a substrate. *Physica A: Statistical Mechanics and its Applications* **1987**, *141* (1), 58-72.
(2) Millero, F. J. The marine inorganic carbon cycle. *Chemical reviews* **2007**, *107* (2), 308-341.
(3) Mucci, A. The solubility of calcite and aragonite in seawater at various salinities, temperatures, and one atmosphere total pressure. *American Journal of Science* **1983**, *283* (7), 780-799. DOI: 10.2475/ajs.283.7.780.
(4) Millero, F. J.; Graham, T. B.; Huang, F.; Bustos-Serrano, H.; Pierrot, D. Dissociation constants of carbonic acid in seawater as a function of salinity and temperature. *Marine Chemistry* **2006**, *100* (1), 80-94. DOI: https://doi.org/10.1016/j.marchem.2005.12.001.
(5) Dickson, A. G.; Riley, J. P. The estimation of acid dissociation constants in seawater media from potentionmetric titrations with strong base. I. The ionic product of water — Kw. *Marine Chemistry* **1979**, *7* (2), 89-99. DOI: https://doi.org/10.1016/0304-4203(79)90001-X.
(6) Press, W. H.; Teukolsky, S. A.; Vetterling, W. T.; Flannery, B. P. *Numerical Recipes 3rd Edition: The Art of Scientific Computing*; Cambridge University Press, 2007.
(7) Wall, T. W.; Greening, D.; Woolsey, R. E. D. OR Practice—Solving Complex Chemical Equilibria Using a Geometric-Programming Based Technique. *Operations Research* **1986**, *34* (3), 345-355.
(8) Hassenkam, T.; Johnsson, A.; Bechgaard, K.; Stipp, S. L. S. Tracking single coccolith dissolution with picogram resolution and implications for CO₂ sequestration and ocean acidification. *Proceedings of the National Academy of Sciences* **2011**, *108* (21), 8571-8576. DOI: doi:10.1073/pnas.1009447108.
(9) Compton, R. G.; Brown, C. A. The inhibition of calcite dissolution/precipitation: Mg2+ cations. *Journal of colloid and interface science* **1994**, *165* (2), 445-449.
(10) Wong, R.; Batchelor-McAuley, C.; Yang, M.; Compton, R. G. The steady-state diffusional flux to isolated square cuboids in solution and supported on an inert substrate. *Journal of Electroanalytical Chemistry* **2021**, *903*, 115818.
